# Therapeutic Potential of Mesenchymal Stem Cells and Their Secretome in the Treatment of Glaucoma

**DOI:** 10.1155/2019/7869130

**Published:** 2019-12-27

**Authors:** C. Randall Harrell, Crissy Fellabaum, Aleksandar Arsenijevic, Bojana Simovic Markovic, Valentin Djonov, Vladislav Volarevic

**Affiliations:** ^1^Regenerative Processing Plant, LLC, 34176 US Highway 19 N Palm Harbor, Palm Harbor, Florida, USA; ^2^Department for Microbiology and Immunology, Center for Molecular Medicine and Stem Cell Research, Faculty of Medical Sciences, University of Kragujevac, 69 Svetozar Markovic Street, Kragujevac, Serbia; ^3^Institute of Anatomy, University of Bern, 2 Baltzerstrasse, Switzerland

## Abstract

Glaucoma represents a group of progressive optic neuropathies characterized by gradual loss of retinal ganglion cells (RGCs), the neurons that conduct visual information from the retina to the brain. Elevated intraocular pressure (IOP) is considered the main reason for enhanced apoptosis of RGCs in glaucoma. Currently used therapeutic agents are not able to repopulate and/or regenerate injured RGCs and, therefore, are ineffective in most patients with advanced glaucoma. Accordingly, several new therapeutic approaches, including stem cell-based therapy, have been explored for the glaucoma treatment. In this review article, we emphasized current knowledge regarding molecular and cellular mechanisms responsible for beneficial effects of mesenchymal stem cells (MSCs) and their secretome in the treatment of glaucoma. MSCs produce neurotrophins and in an exosome-dependent manner supply injured RGCs with growth factors enhancing their survival and regeneration. Additionally, MSCs are able to generate functional RGC-like cells and induce proliferation of retinal stem cells. By supporting integrity of trabecular meshwork, transplanted MSCs alleviate IOP resulting in reduced loss of RGCs. Moreover, MSCs are able to attenuate T cell-driven retinal inflammation providing protection to the injured retinal tissue. In summing up, due to their capacity for neuroprotection and immunomodulation, MSCs and their secretome could be explored in upcoming clinical studies as new therapeutic agents for glaucoma treatment.

## 1. Introduction

Glaucoma, a complex, multifactorial eye disease, is a leading cause of irreversible blindness affecting more than 70 million people worldwide [1]. It represents a group of progressive optic neuropathies characterized by gradual loss of retinal ganglion cells (RGCs), the neurons that conduct visual information from the retina to the brain [2]. An increased production and/or decreased outflow of aqueous humor results in the development of elevated intraocular pressure (IOP) which is considered the main reason for enhanced apoptosis of RGCs in glaucoma [2]. Since RGCs are neurons, their spontaneous regeneration is not feasible, and accordingly, alleviation of IOP and consequent reduction of RGC loss are currently the main approach in glaucoma prevention and therapy [3].

The main target of pharmaceutical and surgical strategies for glaucoma treatment is trabecular meshwork (TM), an outflow system located around the base of the cornea that enables drainage of the aqueous humor [3]. Nevertheless, traditional TM-directed therapies, which downregulate IOP, may only delay progression of glaucoma and are not able to repopulate and/or regenerate RGCs and, therefore, are ineffective in most of patients with advanced glaucoma [1, 3]. Accordingly, several new therapeutic approaches have been investigated for recovering from blindness or for maintenance of remaining vision in glaucoma [4]. Because of their functional properties, mesenchymal stem cells (MSCs) have been the most extensively explored as new therapeutic agents in the cell-based therapy of glaucoma [3–5]. MSCs produce neurotrophins which promote survival and regeneration of injured RGCs in glaucomatous eyes [6]. MSCs are able to repopulate RGCs by generating functional RGC-like cells and by promoting expansion and differentiation of residential retinal stem cells (RSCs) in mature RGCs [7, 8]. Additionally, MSCs may modulate function of TM cells and maintain TM integrity enabling alleviation of IOP in glaucomatous eyes [9].

In this review article, we emphasized current knowledge and future perspectives regarding molecular and cellular mechanisms responsible for beneficial effects of MSCs in the treatment of glaucoma. An extensive literature review was carried out in February 2019 across several databases (Medline, Embase, Google Scholar, and ClinicalTrials.gov), from 1990 to present. Keywords used in the selection were “mesenchymal stem cells”, “glaucoma”, “retinal ganglion cells”, “neurotrophins”, “exosomes”, “retinal stem cells”, and “trabecular meshwork”. All journals were considered, and the initial search retrieved 253 articles. The abstracts of all these articles were subsequently reviewed by three of the authors (CRH, CF, and VV) to check their relevance to the subject of this manuscript. Eligible studies had to delineate molecular and cellular mechanisms involved in the MSC-based therapy of glaucoma, and their findings were analyzed in this review.

## 2. Main Text

### 2.1. Cellular and Molecular Mechanisms Underlying Glaucoma Development

Based on the etiology, glaucoma may be classified into primary glaucoma which develops due to an unknown cause and secondary glaucoma where there is an identifiable cause of increased eye pressure, optic nerve damage, and vision loss (tumor, trauma, pigment dispersion, pseudoexfoliation, and use of corticosteroids) [1].

A genome-wide association study revealed that the two main types of glaucoma (closed-angle and open-angle glaucoma) are distinct genetic entities with different genes associated with each disease [10]. Mutations in collagen type XI alpha 1 chain (COL11A1) and pleckstrin homology domain containing A7 (PLEKHA7) genes were designated as crucially important risk factors for the development of primary closed-angle glaucoma [10–12]. Collagen type XI is structural protein of the trabecular meshwork in the eye while PLEKHA7 protein, expressed in the iris, ciliary body, choroid, and blood-aqueous barrier structures, is involved in paracellular fluid regulation [13, 14]. Accordingly, mutations in COL11A1 and PLEKHA7 genes result in increased accumulation of aqueous humor behind the iris which increases its convexity and causes closure of the angle, site of aqueous outflow in the eye [2, 13, 14]. Accordingly, closed-angle glaucoma is manifested by several symptoms such as blurred vision, sudden sight loss, severe ocular pain, and headache accompanied by nausea or vomiting [15]. Surgical therapy directed at widening the angle and preventing further angle closure is needed for patients suffering from closed-angle glaucoma [15].

In contrast to closed-angle glaucoma, open-angle glaucoma may remain asymptomatic until it results in severe vision impairment [16]. During the early phase, most of patients have complaints related to the loss of peripheral vision manifested as missing words when reading or difficulty with driving. The visual acuity is maintained until late in the disease when apoptotic loss of RGCs significantly impairs patients' ability to perform vision-related activities and reduces quality of life [16].

Mutations of optineurin (OPTN) and myocilin (MYOC) genes were associated with the enhanced apoptosis of RGCs in patients with open-angle glaucoma [10–12]. OPTN protein protects RGCs from apoptosis, and, accordingly, mutations in OPTN gene result in visual impairment due to the enhanced loss of RGCs [2, 12]. Mutations of MYOC gene impair intracellular trafficking resulting in enhanced accumulation of misfolded MYOC protein which provokes an increase of IOP [11, 17]. Elevated IOP, in turn, induces morphological and functional changes in the lamina cribrosa which provides structural and nutrient support to the RGC axons as they leave the eye on their path to the brain [18]. Since RGC axons supply the retina with neurotrophic factors (nerve growth factor (NGF), brain-derived neurotrophic factor (BDNF), ciliary neurotrophic factor (CNTF), and glial cell line-derived neurotrophic factor (GDNF)), glaucomatous changes in the lamina cribrosa result in reduced axonal transport of neurotrophins to RGCs and enhance their apoptotic loss [2, 18].

Since the blood flow in the optic nerve is regulated by endothelial cells and circulating vasoactive factors, a growing body of evidence suggested that vascular dysregulation had an important role in the development and progression of open-angle glaucoma [19–21]. An altered vascular endothelium function, with an imbalance between endothelium-derived vasodilators and vasoconstrictors, has been observed in glaucomatous eyes. Most usually, the level of circulating endothelin-1 (ET-1) was increased in patients who suffer from glaucoma [20]. An elevated level of ET-1 and consequent enhanced activation of ET receptors on smooth muscle cells and pericytes promote influx of calcium and potentiate its liberation from the internal storages. An increased cytoplasmic concentration of calcium induces vasoconstriction which results in reduced and unstable oxygen supply of the optic nerve and consequently led to the development of open-angle glaucoma [21].

It was recently revealed that elevated IOP was involved in the activation of detrimental immune response in glaucomatous eyes [22]. Even transient elevation of IOP may be sufficient to induce enhanced infiltration of activated T cells in the retina. These retina-infiltrating T cells are crucially important for prolonged inflammation and consequent apoptosis of RGCs even when IOP returns to normal [22]. Interestingly, T cells have to be presensitized by commensal bacteria to become activated. The analysis of T cell receptors revealed that most of detrimental T cells were specific for heat-shock proteins (HSPs), alarmins released from injured retinal cells. An increased serum autoantibodies against HSPs and retinal deposits of immunoglobulins were observed in glaucoma patients suggesting the important role of HSPs for the induction of detrimental immune response in glaucomatous eyes [23, 24].

In line with these findings, it is highly expected that therapeutic agents which could protect RGCs from IOP-induced injury and, at the same time, suppress T cell-driven retinal inflammation would be able to efficiently prevent glaucoma progression. Therefore, due to their potential for differentiation in neural lineage cells and capacity for production of neuroprotective and immunosuppressive factors, MSCs were designated as potentially new therapeutic agents in the cell-based therapy of glaucoma.

## 3. MSCs and Their Secretome as New Therapeutic Agents in the Treatment of Glaucoma

MSCs are fibroblast-like, self-renewable cells which reside in almost all postnatal tissues and organs including bone marrow, adipose tissue, blood, umbilical cord, amniotic fluid, dental pulp, and TM [25–28]. In accordance with the criteria set by the International Society for Cellular Therapy, a cell population has to fulfill following criteria to be defined as MSCs: (a) must adhere to plastic in standard culture conditions; (b) must be able to differentiate into adipocytes, osteoblasts, and chondrocytes under standard *in vitro* differentiating conditions; and (c) more than 95% of the cell population must express CD105 (endoglin, involved in proliferation, differentiation, and migration), CD73 (SH3/4, ectoenzyme, regulates the purinergic signaling through the hydrolysis of adenosine triphosphate), and CD90 (Thy-1, regulates differentiation) and must lack expression of CD45, CD34, CD14 or CD11b, CD79a or CD19, and HLA class II, which are membrane markers of leukocytes, thrombocytes, or erythrocytes [29].

Due to their capacity for differentiation into osteoblasts, chondrocytes, and adipocytes, MSCs regulate normal turnover and maintenance of adult mesenchymal tissues [30]. Importantly, several lines of evidence demonstrated that MSCs have a differentiation potential broader than initially thought. Under strictly defined *in vitro* conditions, MSCs could differentiate into cells of neuroectodermal origin, including neuronal cells [30]. Capacity of MSCs to generate neuron-like cells was one of the main reasons why MSCs were designated as new therapeutic agents for cell-based regeneration of RGCs [5]. It was revealed that activation of Wnt/*β*-catenin, Notch, and Sonic-hedgehog pathways as well as inhibition of bone morphogenetic protein 4 (BMP4) signaling in MSCs promoted their differentiation in neuron-like cells [31]. Among growth factors, epithelium growth factor (EGF), basic fibroblast growth factor (bFGF), and hepatocyte growth factor (HGF) have been found effective in inducing generation of neural phenotype in MSCs *in vitro* [31]. *In vivo*, neural differentiation of MSCs was limited by their reduced survival due to the suboptimal availability of neurogenic growth factors [32]. It was recently showed that induction of autophagy in MSCs prior to their transplantation may enhance their survival and differentiation in neuron-like cells *in vivo* [27, 33].

**Figure 1:**
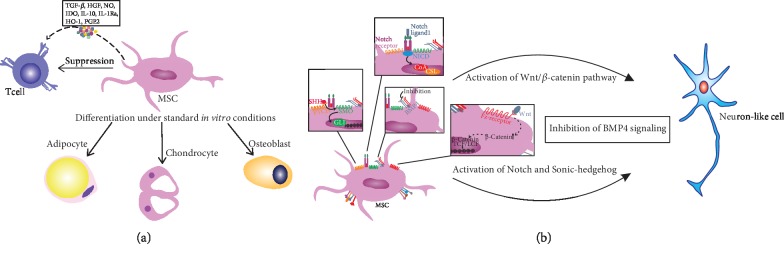
Functional properties of MSCs. MSCs are able to suppress effector T cells in a juxtacrine manner (through the program death (PD) ligand: PD receptor interaction) or in a paracrine manner, via the production of soluble immunoregulatory factors (transforming growth factor-*β* (TGF-*β*), HGF, nitric oxide (NO), indoleamine 2,3-dioxygenase (IDO), interleukin 10 (IL-10), interleukin 1 receptor antagonist (IL-1Ra), heme oxygenase- (HO-) 1, and prostaglandin E2 (PGE2)), and differentiate into adipocytes, osteoblasts, and chondrocytes under standard culture conditions (a). Activation of Wnt/*β*-catenin, Notch, and Sonic-hedgehog pathways as well as inhibition of bone morphogenetic protein 4 (BMP4) signaling in MSCs promoted their differentiation in neuron-like cells (b).

MSCs display a variety of adhesion molecules (C-X-C chemokine receptor type 4 (CXCR-4), CD44, stromal antigen 1 (STAG1), CD166, and CD54/CD102) which enable their migratory and homing characteristics in the injured eye [5]. Due to the high expression of chemokine receptors and adhesion molecules, MSCs become attracted by HSPs, alarmins, and inflammatory chemokines (released from injured RGCs or activated, retinal-infiltrated immune cells), leave their niches, and migrate towards the site of the injury and inflammation which suppress detrimental immune response and promote tissue repair and regeneration [5, 34]. MSCs suppress inflammatory T cells in a juxtacrine manner (through the program death (PD) ligand: PD receptor interaction) or in a paracrine manner, via the production of soluble immunoregulatory factors (transforming growth factor-*β* (TGF-*β*), HGF, nitric oxide (NO), indoleamine 2,3-dioxygenase (IDO), interleukin 10 (IL-10), interleukin 1 receptor antagonist (IL-1Ra), heme oxygenase- (HO-) 1, and prostaglandin E2 (PGE2)) (Figure 1) [35].

However, it should be noticed that MSCs are not constitutively immunosuppressive. Immediately after engraftment in injured tissue, MSCs interact with resident immune cells and, under the influence of local concentration of inflammatory cytokines (TNF-*α*, IL-1*β*, and IFN-*γ*), acquire either pro- or anti-inflammatory properties. Low levels of TNF-*α*, IL-1*β*, and IFN-*γ*, during the early phase of inflammation, induce generation of proinflammatory phenotype in MSCs, which, in turn, through the production of proinflammatory cytokines and chemokines promote influx and activation of circulating phagocytes in injured tissues [5]. Oppositely, MSCs transplanted in the tissue with high concentration of TNF-*α*, IL-1*β*, and IFN-*γ* obtain anti-inflammatory phenotype and suppress activation and effector functions of inflammatory macrophages, DCs, NK and NKT cells, and T lymphocytes, enabling enhanced repair and regeneration of injured tissue [5]. Activation of toll-like receptors (TLRs) has a crucially important role for the generation of immunosuppressive phenotype in MSCs [5]. TLR priming activates phosphoinositide 3-kinase (PI3K)/Akt pathway in MSCs which results in enhanced production of anti-inflammatory cytokines [11]. Activated TLR-2 and TLR-4 recruit PI3K which converts phosphatidylinositol 4,5-bisphosphate (PIP2) to phosphatidylinositol 3,4,5-trisphosphate (PIP3). PIP3 is involved in activation of Akt, which in turn, inactivates Glycogen Synthase Kinase 3 (GSK3) and promotes nuclear accumulation of cAMP Response Element-Binding Protein (CREB) which displaces p65 subunit of nuclear factor kappa-light-chain-enhancer of activated B cells (NF-*κ*B) from the coactivator of transcription (CREB-binding protein (CBP)). An increased transcriptional activity of CREB and consequently reduced transcriptional activity of NF-*κ*B result in increased production of immunosuppressive mediators in MSCs [11]. Among MSC-sourced factors, TGF-*β*, nitric oxide (NO), indoleamine 2,3-dioxygenase (IDO), IL-10, IL-6, leukocyte inhibitory factor (LIF), prostaglandin E2 (PGE2), and IL-1 receptor antagonist (IL-1Ra) have been mainly attributed to the beneficial effects of MSCs in attenuation of acute and chronic inflammation [12].

In line with the above discussed findings, a large number of experimental studies demonstrated that transplantation of MSCs and their secretomes efficiently attenuate glaucoma progression. Beneficial effects of MSCs in the glaucoma treatment mainly relied on their capacity for neurotrophin production, differentiation into functional RGCs, and crosstalk with retinal residential RSCs and TM cells.

## 4. Therapeutic Potential of MSC-Derived Neurotrophins in Glaucoma Treatment

Diverse routes of administration determine whether transplanted MSCs will efficiently engraft and survive in glaucomatous eyes [36]. Injury of RGCs, induced by elevated IOP, is not sufficient to induce breakdown of the blood-retina barrier (BRB) and to enable migration of intravenously (systemically) injected MSCs into the retinas of glaucomatous eyes [36, 37]. On the contrary, the majority of intravitreally transplanted MSCs survive in the vitreous body of glaucomatous eyes several months after injection residing along the inner limiting membrane (ILM) of the retina, the basement membrane of retinal Müller cells [38]. Müller cells change their phenotype and function during glaucoma progression which result in an increased permeability of ILM [39]. Accordingly, some of intravitreally transplanted MSCs may penetrate ILM and migrate in close proximity to the nerve fiber and ganglion cell layers of the retina, which are the most severely damaged in glaucoma [38].

Despite the fact that only a very small amount of transplanted MSCs could incorporate into the injured retinas and differentiate into functional RGCs, significant histological improvement or functional recovery of damaged RGCs was usually detected after the intravitreal administration of MSCs [38]. Accordingly, several lines of evidence demonstrated that MSCs, in a paracrine manner, promote survival and endogenous repair of injured RGCs. MSCs are capable to produce large amounts of neurotrophic factors (NGF, EGF, bFGF, BDNF, CNTF, and platelet-derived growth factor (PDGF)) which may increase proliferation and differentiation of retinal progenitor cells, and most of them were able to efficiently prevent apoptosis and promote survival of RGCs in glaucomatous eyes [2].

After engraftment in injured retinas, MSCs produce NGF and BDNF and promote regeneration of RGCs [2, 40, 41]. NGF is a neurotrophic factor involved in the development, maintenance, and regeneration of mammalian neurons, having protective effects in optic neuropathies, including glaucoma [41]. NGF induces enhanced expression of antiapoptotic Bcl-2 protein and attenuates expression of proapoptotic Bax protein in RGCs [42]. Compared to healthy controls, serum levels of NGF were significantly lower in glaucoma patients [43], indicating that downregulated production of NGF might contribute to the enhanced loss of RGCs. In line with these observations, Lambiase and colleagues observed long-lasting improvements in the visual field, optic nerve function, contrast sensitivity, and visual acuity in glaucoma patients treated with NGF eye drops, demonstrating beneficial and neuroprotective effects of exogenously administrated NGF in glaucoma therapy [42]. BDNF is a neurotrophin which is increasingly expressed in the retinas of glaucomatous eyes [2]. Binding of BDNF to its receptors (tropomyosin receptor kinase B (TrkB) and the pan-neurotrophin p75^NTR^) induces activation of c-jun and suppression of caspase-2, which prevents apoptosis and promotes survival of RGCs [36]. The serum level of BDNF was significantly lower in glaucoma patients compared to healthy controls, and decreased production of BDNF correlated with reduced survival of RGCs [43]. Nevertheless, effective delivery of exogenous NTF and BDNF to the glaucomatous eye is limited by several obstacles including the downregulation of TrkB receptors after bolus administration [44–46]. Mead and coworkers addressed these problems by intravitreal transplantation of dental pulp-derived MSCs (DP-MSCs) which are engrafted in close proximity to the retina and continuously delivered NTF and BDNF to injured RGCs promoting their survival and regeneration [47, 48].

Similarly, Johnson and colleagues demonstrated that intravitreal administration of MSCs efficiently suppressed apoptosis and promoted survival of RGCs in a PDGF- and CNTF-dependent manner [49]. MSC-derived PDGF induced phosphorylation and activation of signal transducer and activator of transcription 3 (STAT-3) in RGCs which downregulated the expression of proapoptotic Bax and consequently reduced apoptotic loss of RGCs [6]. Anti-PDGF treatment prevented the expression of STAT-3 and completely inhibited MSC-based suppression of Bax in RGCs [6], indicating an important role of MSC-derived PDGF for survival of RGCs [6, 49]. In a similar manner as PDGF, MSC-derived CNTF reduced apoptotic loss of RGCs in glaucomatous eyes by promoting phosphorylation of cytoplasmic STAT-3 [50]. Nevertheless, it has to be highlighted that significant visual impairment was noticed in healthy rat eyes injected with a high dose of CNTF [51], suggesting that intraocular levels of CNTF need to be continuously monitored and controlled during MSC-based therapy to prevent detrimental effects of high concentration of CNTF on vision function.

In line with these findings, several lines of evidence demonstrated that beneficial effects of neurotrophins were dependent on their continuous release [52–54]. Since MSCs home to the site of retinal injury and are able to produce NGF, BDNF, CNTF, and PDGF in an injury-dependent manner, these stem cells were used as vehicles for continuous delivery of neurotrophins in the glaucoma treatment [36]. Harper and colleagues engineered BDNF-overexpressing MSCs (MSCs^BDNF^). Intravitreally transplanted MSCs^BDNF^ better survived in glaucomatous rat eyes and provided significantly improved functional and structural protection to RGCs than genetically nonmodified MSCs [40], indicating therapeutic potential of MSCs^BDNF^ in cell-based treatment of glaucoma. A similar approach was used by Harrell and coworkers who engineered NGF-overexpressing MSCs (MSCs^NGF^) [5]. Intravitreally injected MSCs^NGF^ were successfully engrafted in the injured retinas and promoted RGC survival and regeneration in a NGF-dependent manner, indicating therapeutic potential of MSCs^NGF^ in glaucoma therapy [55].

## 5. MSC-Derived Exosomes as Nanocarriers for Neurotrophin Delivery to the Injured RGCs

MSCs hold great potential in regenerative ophthalmology due to their capacity for secretion of exosomes (Exos), membrane enclosed, nanosize (30-100 nm), extracellular vesicles that contain messenger ribonucleic acid (mRNA), microRNA (miRNA), and proteins, including neurotrophins [5]. MSC-derived Exos (MSC-Exos) are able to reside in the vitreous humor at least for four weeks after intravitreal administration and, due to their nanodimension, may rapidly reach RGCs to supply them with neurotrophins [56]. As demonstrated by Mead and Tomarev [57], cell death of RGCs was significantly reduced in animals treated with BM-MSC-Exos. By delivering BDNF, NGF, and PDGF to RGCs, BM-MSC-Exos provided neuroprotection which significantly reduced the total number of degenerating axons in optic nerves of glaucomatous eyes [58]. Importantly, these beneficial effects were observed only in animals that received MSC-derived Exos and were not noticed after intravitreal injection of fibroblast-derived Exos, indicating specific therapeutic potential of MSC-Exos in RGC regeneration and glaucoma treatment [59].

Interestingly, therapeutic effects of BM-MSC-Exos were significantly better than those obtained after transplantation of BM-MSCs. BM-MSCs lack the capacity to integrate into the retina and remain in the vitreous body after injection [37, 57]. On the contrary, BM-MSC-Exos diffused rapidly throughout the retina, and within one hour after injection, intravitreally administered BM-MSC-Exos were able to successfully deliver neurotrophins to the injured RGCs promoting their survival and regeneration [5, 56, 57]. However, it has to be noted that therapeutic efficacy of BM-MSC-Exos was only observed when BM-MSC-Exos were intravitreally injected every week or every month. Longer delays between treatments completely abrogated MSC-Exo-dependent effects, suggesting that their beneficial effects were temporary and relied on their repetitive injection [59]. On the contrary, BM-MSCs remain in the glaucomatous eyes months after intravitreal injection and, accordingly, may provide long-lasting neuroprotection due to the continuous release of neurotrophins.

MSC-Exos elicited their therapeutic effects through miRNA-dependent mechanisms. It was shown that knockdown of Argonaute2 protein, which is crucially important for miRNA function, significantly attenuated BM-MSC-Exo-induced effects [59]. RNA sequencing revealed that more than 40 miRNAs were upregulated in BM-MSC-Exos, compared to fibroblast-derived Exos, and among them, miR-17-92, miR-21, and miR146a were designated as the most important for regeneration of RGCs in glaucomatous eyes [5, 57, 58]. The expression of phosphatase and tensin homolog (PTEN), which is an important suppressor of RGC axonal growth and survival, was regulated by miR-17-92 and miR-21 while miR-146a modulated expression of epidermal growth factor receptor (EGFR) involved in inhibition of axon regeneration [5].

## 6. Dental Pulp and Amniotic Fluid as Valuable and Easily Accessible Sources for MSC-Based Therapy of Glaucoma

**Figure 2:**
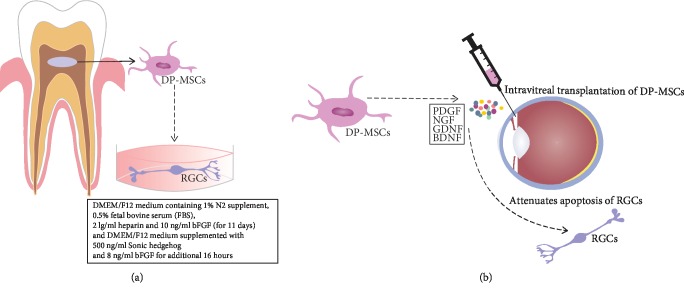
Therapeutic potential of DP-MSCs in glaucoma treatment. Dental pulp represents valuable and easily accessible sources for DP-MSCs which are able to differentiate into functional retinal ganglion cells (RGCs) under appropriate culture conditions (a). Intravitreally transplanted DP-MSCs produce several neurotrophic factors (platelet-derived growth factor (PDGF), nerve growth factor (NGF), brain-derived neurotrophic factor (BDNF), and glial cell line-derived neurotrophic factor (GDNF)) which promote survival of RGCs and induce regeneration of injured axons (b).

MSCs develop distinct functional characteristics in response to the microenvironment to which they are exposed [60]. Accordingly, Mead and colleagues compared human DP-MSCs, bone marrow-derived MSCs (BM-MSCs), and adipose tissue-derived MSCs (AT-MSCs) for their potential to promote regeneration of injured RGCs in glaucomatous eyes [61]. Among compared subpopulations of human MSCs, DP-MSCs produced the largest amounts of neurotrophic factors (PDGF, NGF, GDNF, and BDNF) and most efficiently protected RGCs against apoptosis (Figure 2). Similar findings were obtained *in vivo*, in the animal model of RGC injury where intravitreally transplanted DP-MSCs promoted a significantly greater increase in RGC survival and significantly higher increase in the number of regenerating axons compared with similarly injected BM-MSCs [47]. Based on all these results, dental pulp has been proposed as a valuable and easily accessible source of DP-MSCs which could be used in autologous cell-based therapy of glaucoma [48].

In line with these findings, Roozafzoon and colleagues induced differentiation of DP-MSCs into functional RGCs by using RGC differentiation medium and a three-dimensional fibrin network as an environment which mimicked mechanical properties of the native retina [7]. Isolated DP-MSCs were initially differentiated on 150 lg/ml poly-D-Lysine and 1 lg/ml Laminin substrate for 11 days in a differentiation Dulbecco's modified Eagle medium/Nutrient Mixture F-12 (DMEM/F12) medium containing 1% N2 supplement, 0.5% fetal bovine serum (FBS), 2 lg/ml heparin, and 10 ng/ml bFGF. Afterwards, the cells were grown in DMEM/F12 medium supplemented with growth factors (500 ng/ml Sonic-hedgehog (Shh) and 8 ng/ml bFGF) for additional 16 hours. Immunocytochemical and gene expression analysis revealed increased expression of RGC specific transcriptional factors (Pax6, Atoh7, and BRN3B) in differentiated cells [7], indicating successful differentiation of DP-MSCs in RGC-like cells.

In addition to dental pulp, amniotic fluid also contains a variety of growth factors that are crucial for the development of RGCs [62]. Additionally, amniotic fluid serves as a rich and advantageous source of amniotic fluid-derived MSCs (AF-MSCs) which, similar as DP-MSCs, exhibited greater capacity for cell proliferation, self-renewal, and differentiation in neural cells than BM-MSCs [63, 64]. AF-MSCs more rapidly formed neurospheres *in vitro*, showed higher expression of neural stemness markers following neural stem cell differentiation (Nestin, vimentin, and Musashi), and have higher capacity for production of BDGF and NGF [64].

It was recently revealed that Exos derived from AF-MSCs contain immunosuppressive factors TGF-*β* and HGF which suppress proliferation of activated T cells by causing the G1 cell cycle arrest [65–67]. Interestingly, AF-MSC-Exos selectively downregulated Janus kinase/Stat signaling pathways in inflammatory T cells without affecting expansion and immunosuppressive properties of CD4+CD25+FoxP3+ T regulatory cells [68], indicating their therapeutic use in the treatment of T cell-driven inflammatory diseases, including glaucoma. In line with these findings, we recently developed neuroprotective and immunomodulatory ophthalmic solution (“Exosome-Derived Multiple Allogeneic Protein Paracrine Signaling (Exosomes D-MAPPS)”) which activity is based on the capacity of AF-MSC-derived exosomes to produce neurotrophins (PDGF, NGF) and immunomodulatory factors (TGF-*β*, HGF) enabling tissue repair in neurodegenerative and inflammatory eye diseases [5, 67, 68].

## 7. Crosstalk between MSCs and Residential Retinal Cells in MSC-Based Alleviation of Glaucoma

Although it was well documented that MSC-derived neurotrophins were responsible for beneficial effects of transplanted MSCs on survival of injured RGCs [52–54], recently published data indicated that interaction between transplanted MSCs and residential retinal cells also contributes to the MSC-based alleviation of glaucoma [5, 31–33].

Several lines of evidence demonstrated that dysfunction and degeneration of TM are the main factors responsible for the development of elevated IOP in glaucomatous eyes [69–74]. Apoptosis and oxidative stress induce a reduced number of TM cells while TGF-*β*-driven fibrosis provokes remodeling of the extracellular matrix and induces an increase in aqueous humor outflow resistance that led to the elevation of IOP and consequent development of glaucoma [71–74]. Accordingly, therapeutic agents which support TM integrity could prevent increase of IOP and alleviate glaucoma progression. Interestingly, MSCs and TM cells shared several phenotypic and functional characteristics [75]. Similar to MSCs, TM cells expressed CD73, CD90, CD105, and CD146 and lack expression of CD31, CD34, and CD45. Additionally, the expression of transcriptional factors responsible for cell potency and proliferation (sox2 and notch1) was detected in TM cells as it was observed in DP-MSCs and AF-MSCs [75].

In addition to this data, Tay and colleagues managed to isolate MSCs from TM area (TM-derived MSCs; TM-MSCs) [28]. TM-MSCs had spindle-shaped morphology, express CD73, CD90, CD105, and CD146, and were capable to differentiate into adipocytes, chondrocytes, and osteocytes [28]. TM-MSCs also express Ankyrin3, Low-Density Lipoprotein Receptor, Chitinase3-Like-1, Human Milk Fat Globule 1, Matrix Metalloproteinase 1 (MMP1), and Aquaporin 1 which are present in mature TM cells, suggesting that TM-MSCs are progenitors of the meshwork tissue which are able to efficiently replace injured TM cells. Accordingly, isolation of TM-MSCs from glaucoma patients and their subsequent autologous transplantation represent a potentially new therapeutic strategy for cell-based therapy of glaucoma that should be explored in future experimental and clinical studies [28].

Importantly, it should be highlighted that mature, fully differentiated TM cells have different phenotypic and functional properties than TM-MSCs. In contrast to TM-MSCs, mature TM cells could not differentiate into adipocytes or osteocytes and had high expression of *α*-smooth muscle actin, myocilin, and angiopoietin-like 7 which regulate TM integrity and collagen synthesis [28, 75]. Accordingly, several research groups investigated whether MSCs and/or their secretomes alleviated glaucoma by regulating function of mature TM cells [9, 76, 77]. Roubeix and colleagues demonstrated that injection of BM-MSCs in the anterior chamber of hypertensive rat eyes significantly attenuated IOP by modulating function of TM cells in a paracrine manner [9]. The significant increase in the total number of functional RGCs was noticed in glaucomatous eyes that received either MSCs or MSC-derived conditioned medium (MSC-CM). MSC-CM-based therapy managed to activate antiapoptotic pathways in TM cells in an Akt-dependent manner and induced decreased myosin phosphorylation which resulted in relaxation of TM cells, reduced aqueous humor outflow resistance, and attenuated IOP. Moreover, MSC-CM suppressed activation of TGF-*β* signaling and attenuated collagen production in TM cells [9]. Similar results were reported by Manuguerra-Gagné and colleagues who demonstrated that injection of MSCs and their secretomes completely restored TM functionality in laser-treated eyes [76]. Interestingly, attenuated IOP were only observed in glaucomatous eyes that received low-oxygen pretreated MSCs while no significant effect on glaucoma progression was observed after injection of normoxic MSCs [76]. Preconditioning by brief hypoxia provoked enhanced expression of hypoxia-inducible factor 1 (HIF-1) in MSCs [27]. HIF-1 is a master regulator of adaptive responses to hypoxia that activates autophagy-related mitogenic neuropeptide Apelin and prevents apoptosis. Accordingly, enhanced expression of HIF-1 in low-oxygen pretreated MSCs enabled their better adaptation to hypoxia-induced stress [27, 77]. These MSCs were capable to better engraft and survive in the TM area in a paracrine manner, modulating TM integrity by suppressing collagen synthesis in TM cells as well as through the activation of neural progenitors and residential RSCs [76].

RSCs, which generate all the neurons of the mature retina, reside in the ciliary margin area of the retina and, therefore, were designated as pigmented cells from the ciliary margin (PCMs) [78]. The total number of RSCs in adult mammalian eyes is insufficient for optimal regeneration of RGCs in glaucomatous eyes [79]. Since the ciliary margin area is very small and proliferation ability of PCMs is limited, only a small number of patients' own PCMs may be isolated and used for autologous transplantation [78, 80]. Recently, Li and colleagues revealed that coculture of PCMs with BM-MSCs significantly increased the proliferation rate of PCMs and notably enhanced their differentiation in functional RGCs [8]. Major markers of retinal differentiation, including rhodopsin, visual system homeobox 2, heparin sulfate, and photoreceptor-specific homeobox gene (cone-rod homeobox, Crx), were remarkably upregulated in PCMs following coculture with BM-MSCs, indicating that BM-MSC-based priming of PCMs should be further explored as a new approach which could enable autologous transplantation of RGCs in glaucoma patients [8].

## 8. Clinical Use of MSCs in the Therapy of Glaucoma

Although encouraging results related to the MSC-based therapy of glaucoma were obtained in a large number of preclinical studies, there is still no evidence that transplantation of MSCs may repopulate RGCs, attenuate IOP, and restore visual function in patients suffering from glaucoma. Three clinical studies (NCT02330978, NCT01920867, and NCT03011541) were aimed at demonstrating safety and efficacy of MSC-based therapy in glaucoma, but none of them have been completed yet.

De Paula and colleagues planned to investigate whether intravitreal transplantation of autologous BM-MSCs could show beneficial effects in patients suffering from advanced glaucoma (NCT02330978). Although the estimated study completion date was December 2016, obtained results are still not published and the current status of this study is unknown.

Two other clinical trials (NCT01920867 and NCT03011541) will be conducted by Weiss and coworkers who are going to evaluate the use of autologous BM-derived stem cells for the treatment of retinal and optic nerve diseases, including glaucoma. BM-derived stem cells will be delivered by different routes (intravitreally, intravenously, and intraocular with vitrectomy prior to intraocular injection which may result in a larger amount of stem cells in the intravitreal cavity). Patients will be followed for one year with serial comprehensive eye examinations including imaging and diagnostic ophthalmic testing. Since these studies still recruit patients and estimated study completion dates are August 2019 (for NCT01920867) and January 2021 (for NCT03011541), it is expected that obtained results will be published in upcoming years.

## 9. Conclusions

In summing up, results obtained in a large number of experimental studies revealed that beneficial effects of MSCs and their secretome in glaucoma therapy relied on their capacity for neuroprotection and RGC regeneration. Through the production of neurotrophins and vasoactive and immunomodulatory factors, MSCs induce expansion and regeneration of RGCs, provide maintenance of TM integrity, and attenuate retinal inflammation in animal models of glaucoma (Table 1). Nevertheless, it has to be highlighted that therapeutic potential of MSCs and their secretome in the treatment of glaucoma has not been validated in clinical settings yet. The optimal origin, number, and route of administration as well as safety of MSC-based therapy still have to be determined in clinical trials with the appropriate number of enrolled patients. Accordingly, the conclusion that MSCs and their secretome represent new human remedy for the treatment of glaucoma can be made only if MSC-dependent therapeutic effects will be confirmed in future clinical trials.

## Figures and Tables

**Table 1:** Molecular mechanism responsible for beneficial effects of MSCs and their secretome in glaucoma treatment.

Type of MSCs or their secretome	Target cell	MSC-derived factor	Mechanism of action	Effect	Ref.
DP-MSCs; MSCs^NGF^	RGCs	NGF	Enhanced expression of Bcl-2 and attenuated expression of Bax in RGCs	Reduced apoptosis of RGCs	[5, 47, 48]
DP-MSCs; MSCs^BDNF^	RGCs	BDNF	Activation of c-jun and suppression of caspase-2 in RGCs	Increased survival of RGCs	[40, 47, 48]
BM-MSCs	RGCs	PDGF; CNTF	Increased phosphorylation and activation of STAT-3	Reduced apoptotic loss of RGCs	[6, 50]
BM-MSC-Exos	RGCs	BDNF; NGF; PDGF	Trophic support; miR-17-92 and miR-21-dependent suppression of PTEN	Increased regeneration of RGCs	[5, 56–58]
AF-MSC-Exos	T cells	TGF-*β*; HGF	Suppression of Janus kinase/Stat pathways in inflammatory T cells	Attenuated inflammation in glaucomatous eyes	[5, 67, 68]
TM-MSCs; BM-MSC; BM-MSC-CM	TM cells	sox2; notch1; HIF-1	Activation of autophagy-related mitogenic neuropeptide Apelin; decreased myosin phosphorylation; replacement of injured TM cells	Reduced apoptosis of TM cells; relaxation of TM cells; reduced aqueous humor outflow resistance; attenuated IOP	[9, 75, 76]

Abbreviations: DP-MSCs: dental pulp-derived MSCs; NGF: nerve growth factor; MSCs^NGF^: NGF-overexpressing MSCs; RGCs: retinal ganglion cells; BDNF: brain-derived neurotrophic factor; MSCs^BDNF^: BDNF-overexpressing MSCs; BM-MSCs: bone marrow-derived MSCs; PDGF: platelet-derived growth factor; CNTF: ciliary neurotrophic factor; STAT-3: signal transducer and activator of transcription 3; Exos: exosomes; PTEN: phosphatase and tensin homolog; AF-MSCs: amniotic fluid-derived MSCs; TGF-*β*: transforming growth factor-*β*; HGF: hepatocyte growth factor; MSC-CM: MSC-derived conditioned medium; HIF-1: hypoxia-inducible factor 1; IOP: intraocular pressure.
